# Chemical, Physical, and Mechanical Properties of Belangke Bamboo (*Gigantochloa pruriens*) and Its Application as a Reinforcing Material in Particleboard Manufacturing

**DOI:** 10.3390/polym14153111

**Published:** 2022-07-30

**Authors:** Apri Heri Iswanto, Elvara Windra Madyaratri, Nicko Septuari Hutabarat, Eka Rahman Zunaedi, Atmawi Darwis, Wahyu Hidayat, Arida Susilowati, Danang Sudarwoko Adi, Muhammad Adly Rahandi Lubis, Tito Sucipto, Widya Fatriasari, Petar Antov, Viktor Savov, Lee Seng Hua

**Affiliations:** 1 Department of Forest Product, Faculty of Forestry, Universitas Sumatera Utara, Kampus USU Padang Bulan, Medan 20155, Indonesia; nickohutabarat0709@gmail.com (N.S.H.); ekazunaidi99@gmail.com (E.R.Z.); titomedan@yahoo.com (T.S.); 2 JATI-Sumatran Forestry Analysis Study Center, Faculty of Forestry, Universitas Sumatera Utara, Kampus USU Padang Bulan, Medan 20155, Indonesia; 3 Department of Forest Products, Faculty of Forestry and Environment, IPB University, Bogor 16680, Indonesia; elvarawindra@yahoo.com; 4 Research Center for Biomass and Bioproducts, National Research and Innovation Agency, Jl Raya Bogor KM 46, Cibinong 16911, Indonesia; danang@biomaterial.lipi.go.id (D.S.A.); marl@biomaterial.lipi.go.id (M.A.R.L.); widya.fatriasari@biomaterial.lipi.go.id (W.F.); 5 School of Life Sciences and Technology, Institut Teknologi Bandung, Gedung Labtex XI, Jalan Ganesha 10, Bandung 40132, Indonesia; atmawidarwis@gmail.com; 6 Department of Forestry, Faculty of Agriculture, University of Lampung, Jl Sumantri Brojonegoro, Gedung Meneng, Bandar Lampung 35145, Indonesia; wahyu.hidayat@fp.unila.ac.id; 7 Department of Silviculture, Faculty of Forestry, Universitas Sumatera Utara, Kampus USU Padang Bulan, Medan 20155, Indonesia; arida.susilowati@usu.ac.id; 8 Research Collaboration Center for Biomass and Biorefinery between BRIN, Universitas Padjadjaran, National Research and Innovation Agency, Jl Raya Bogor KM 46, Cibinong 16911, Indonesia; 9 Faculty of Forest Industry, University of Forestry, 1797 Sofia, Bulgaria; victor_savov@ltu.bg; 10 Laboratory of Biopolymer and Derivatives, Institute of Tropical Forestry and Forest Product, Universiti Putra Malaysia, Kuala Lumpur 43400, Malaysia; lee_seng@upm.edu.my

**Keywords:** *Gigantochloa pruriens*, chemical properties, physical and mechanical properties, wood-based composites, particleboard

## Abstract

This study aimed to analyze the basic properties (chemical composition and physical and mechanical properties) of belangke bamboo (*Gigantochloa pruriens*) and its potential as a particleboard reinforcement material, aimed at increasing the mechanical properties of the boards. The chemical composition was determined by Fourier transform near infrared (NIR) analysis and X-ray diffraction (XRD) analysis. The physical and mechanical properties of bamboo were evaluated following the Japanese standard JIS A 5908 (2003) and the ISO 22157:2004 standard, respectively. The results showed that this bamboo had average lignin, holocellulose, and alpha-cellulose content of 29.78%, 65.13%, and 41.48%, respectively, with a degree of crystallinity of 33.54%. The physical properties of bamboo, including specific gravity, inner and outer diameter shrinkage, and linear shrinkage, were 0.59%, 2.18%, 2.26%, and 0.18%, respectively. Meanwhile, bamboo’s mechanical properties, including compressive strength, shear strength, and tensile strength, were 42.19 MPa, 7.63 MPa, and 163.8 MPa, respectively. Markedly, the addition of belangke bamboo strands as a reinforcing material (surface coating) in particleboards significantly improved the mechanical properties of the boards, increasing the modulus of elasticity (MOE) and bending strength (MOR) values of the fabricated composites by 16 and 3 times.

## 1. Introduction

As a country known for its high biodiversity, Indonesia has hundreds of bamboo species. It has the third-largest bamboo population in the world after China and India. Bamboo grows in dry to wet, tropical climates [1,2,3,4]. In addition to being used as a nonpermanent material in construction and furniture making, it has various industrial applications, such as fiber reinforcement, paper, textiles, oriented strand board, particleboard, food, and bioenergy [5,6,7,8,9,10]. Furthermore, it is a fast-growing plant with a short harvest time (3–5 years old) and high productivity of 20–40 tons/ha/year, 7–30% higher than woody plants [5,11,12,13].

One of the most common bamboo species of the *Gigantochloa* genus, found in northern Sumatra, is the *Gigantochloa pruriens*, an endemic species spreading across the Karo and Gayo districts in Indonesia. The culms of *Gigantochloa pruriens* are regarded as valuable feedstocks widely used in construction applications for manufacturing walls, pillars, roofs, etc. Similar to wood, bamboo also has various properties. The characterization of chemical, physical, and mechanical properties of belangke bamboo raw materials is a very important factor in determining its suitability for various commercial applications, one of which is as a raw material for manufacturing particleboard. Currently, research data related to belangke bamboo is still rather limited. Based on the literature review results, only one study related to the anatomical properties of belangke bamboo was found, which was reported by Darwis et al. [14]. Several studies have shown that, in general, bamboo has good mechanical properties so that it can be used as a raw material for structural composites and as a surface layer modification material to improve the strength of particleboard [7,9,10,15,16,17].

In particleboard manufacturing, mixing particles of two different species is not a common practice. Wood particles with stronger mechanical strength could be used to compensate for the inferiority of the other weaker wood particles in the particleboard [18,19]. Lee et al. [18,19] used rubberwood (*Hevea brasiliensis*) particles as a surface layer and oil palm trunk particles as a core layer to produce three-layer particleboard. The mechanical properties of the particleboard were found to increase along with the increased proportion of rubberwood particles in the surface layer. The studies demonstrated that the employment of stronger wood species as surface layer could result in improved mechanical strength of the composites. Bamboo was found to be a good reinforcing material for particleboard. De Almeida et al. [20] reported that the incorporation of 25% and 50% of *Dendrocalamus asper* bamboo into particleboard made of *Eucalyptus urophylla* × *grandis* wood had improved the bending strength of the particleboard produced. Mixing a more compressible wood with other noncompressible wood could enhance the compression and consolidation of the particleboard [21]. A study by Zaia et al. [22] reported the application of bamboo laminas of *Dendrocalamus giganteus* as a reinforcement for particleboard. The board produced showed a great potential to be used as construction materials. However, relevant studies are scarce, particularly those involving bamboo strands. Therefore, the goal of this research work was to investigate the basic properties (chemical composition, physical, and mechanical properties) of belangke bamboo (*Gigantochloa pruriens*) and evaluate its potential as a particleboard reinforcing material, aimed at improving the boards’ mechanical properties.

## 2. Materials and Methods

### 2.1. Materials

**Figure 1:**
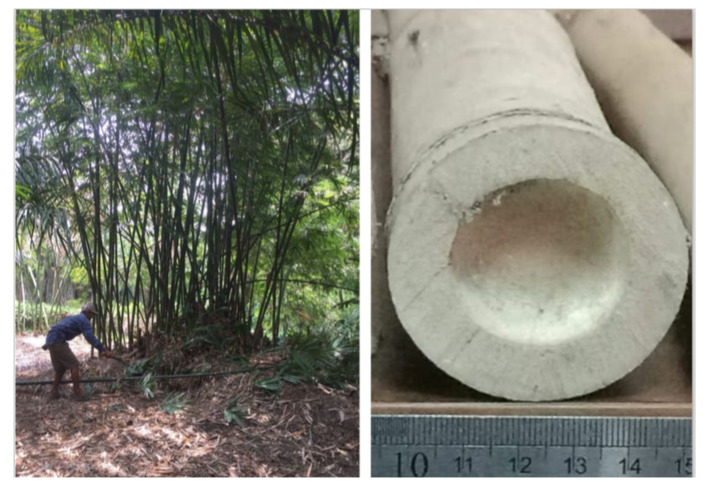
Bamboo clumps and bamboo middle culm cross-sections.

The *Gigantochloa pruriens* bamboo (Figure 1) was obtained from the Binjai region, North Sumatra (3°35′55″ N, 98°28′49″ E). The length and diameter of belangke bamboo used in this work were approximately 14 m and 6 cm, respectively.

**Figure 2:**
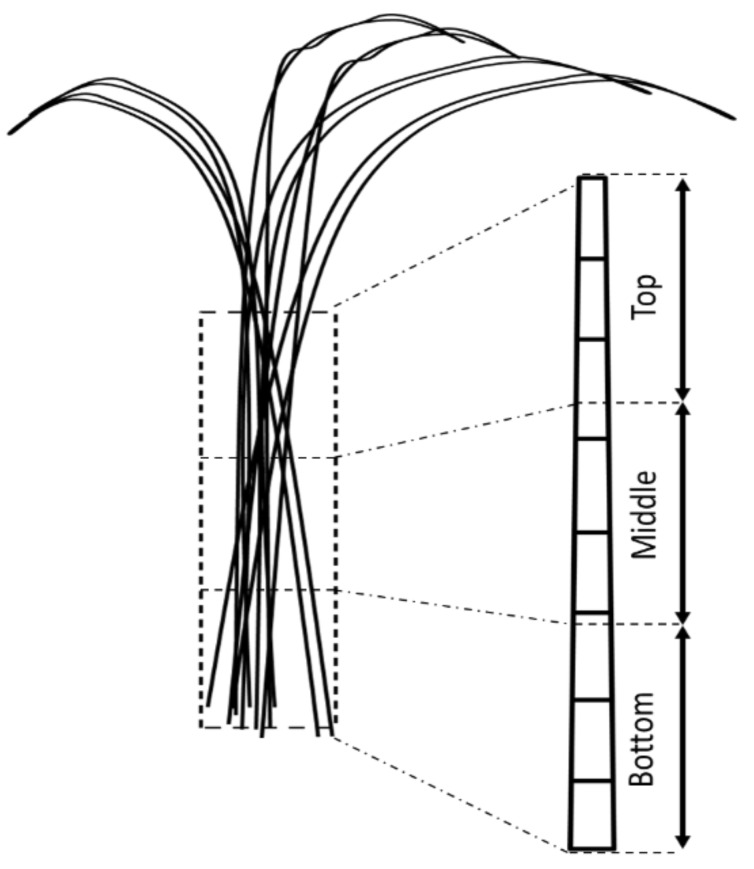
Bamboo culm parts (bottom, middle, and top) for analysis of chemical components and physical–mechanical properties of bamboo.

Furthermore, the samples used for chemical and crystallinity composition observation were divided into three parts, namely bottom (B), middle (M), and top (T) as presented in Figure 2.

### 2.2. Methods

#### 2.2.1. Chemical Component Analysis

##### Material Preparation

Sample preparation was performed according to the standards of the Technical Association of the Pulp and Paper Industry (TAPPI) T 257 cm-02 [23] and T 264 cm-97 [24]. Furthermore, samples were mashed using a ring flaker/hammer mill/disk mill until all filtered through a sieve, number 40 mesh.

##### Determination of Chemical Components

- Acid Insoluble Lignin Contents

This test was performed based on National Renewable Energy Laboratory (NREL) Laboratory Analytical Procedure (LAP) 003 standard [25]. Empty filter funnel 1G3 was dried in an oven at 105 °C for at least 4 h before testing, cooled in a desiccator for 30 min, and weighed in the dry weight of the oven. Furthermore, a total of 0.3 g of the extractive free sample was weighed and put in a small vial with a wide mouth of ±20 mL, and the water content was measured based on TAPPI T 264 cm-97. In addition, it was added to 3 mL of 72% sulfuric acid (Merck, Darmstadt, Germany), stirred using a magnetic stirrer for 2 h with 320 rpm at room temperature (conditioned using a Petri dish filled with water), transferred to a 100 mL Duran bottle, and diluted using 84 mL of distilled water to a final concentration of 4% sulfuric acid (Merck). The sample was tightly closed and heated by autoclaving at 121 °C for 1 h. Furthermore, a total of ±10 mL was filtered using an IG3 glass filter with the help of a vacuum and stored for measurement of ASL. The samples in the IG3 filter funnel were washed with a minimum of 50 mL of hot water and dried in an oven at 105 °C for 24 h. Consequently, the sample was removed from the oven and cooled in a desiccator for 30 min, and weighed.

b. Acid Soluble Lignin Contents

This test was performed based on the NREL LAP 003 standard [25]. The required volume of hydrolyzed filtrate sample and sulfuric acid (Merck) were diluted in a centrifuge tube or other container and was used to blank the spectrophotometer (Shimadzu UV-1800, Kyoto, Japan), which was set at a wavelength of 205 nm. The absorbance was measured within the range of 0.2–0.8 AU (absorption units). An absorbance outside this range indicates that the dilution should be performed with a blank of sulfuric acid (Merck).

c. Holocellulose Contents

A 1G3 filter funnel was dried in an oven at 105 °C for at least 4 h before testing. Furthermore, it was cooled in a desiccator for 30 min, and the oven-dry weight was weighed. A total of 1.0 g of the extractive-free sample was weighed and put into a 100 mL Erlenmeyer. Simultaneously, the sample’s water content was measured, and 40 mL of aquadest was added. A total of 1.5 mL of 25% sodium chlorite (Merck) was added to 0.125 mL of 100% glacial acetic acid (Merck), tightly closed using heat-resistant plastic, and tightly tied using a rubber band. It was heated in a water bath for 1 h at 80 °C and repeated three times, cooled in an ice bath, and filtered using a previously weighed IG3 filter funnel. In addition, it was washed with 100 mL of cold water and 25 mL of acetone and dried in an oven at 105 °C for 24 h. Consequently, it was removed from the oven and cooled in a desiccator for 30 min, and weighed.

d. Alpha Cellulose Contents

The test was performed using an empty 1G3 filter funnel dried in an oven at 105 °C for at least 4 h before testing. The IG3 was cooled in a desiccator for 30 min, and the oven-dry weight was weighed. Furthermore, a total of 0.5 g of the holocellulose sample was placed into a ±20 mL wide-mouth vial. Subsequently, the water content of the sample was measured, and 6.25 mL of 17% sodium hydroxide (Merck) was added, ensuring that all samples had been moistened with the solvent. It was stirred using a magnetic stirrer for 15 min at 320 rpm and left without stirring for 30 min. Afterward, 8.25 mL was added, stirred for 5 min with 320 rpm, and left without stirring for 1 h. The sample was filtered using an IG3 filter glass, rinsed using 25 mL of 8.3% sodium hydroxide (Merck), and washed with 100 mL of aquadest. Furthermore, it was added to 10 mL of 10% acetone (technical) and soaked for 3 min, rinsed with aquadest until the pH became neutral, and dried in an oven at 105 °C for 24 h. Afterward, it was removed from the oven and cooled in a desiccator for 30 min, and weighed.

e. Extractive Contents in Ethanol Benzene (1:2)

This test was performed based on the standard TAPPI T 204 cm-97 [24] by drying the boiling flask in an oven at 105 °C for at least 4 h and cooling it in a desiccator for 30 min. A total of 3 g oven-dry weight (record the weight) of the empty boiling flask was weighed, inserted, and wrapped in filter paper with cotton at both ends and tied using mattress thread. The sample’s moisture content was calculated simultaneously at the time of weighing the sample for extractives. Meanwhile, the extractive device was arranged and turned on, adding 150 mL of 150 mL ethanol–benzene (1:2) into a boiling flask. It was extracted for a period of 24 cycles for 4–5 h until all were dissolved in the extracting solution (marked by the extracting solution in a colorless soxhlet) and removed from the soxhlet. Furthermore, the extract solution in the flask was boiled in an oven at 40 °C and stored for further testing (holocellulose and lignin), evaporated until about 5 mL was left, and dried in an oven at 105 °C for 24 h. Afterward, it was cooled in a desiccator for 30 min and weighed.

f. Ash Contents

This test was performed based on TAPPI T 211 cm-02 standard [26]. The crucible porcelain was dried in a furnace (Blue M, New Columbia, United States) for 30–60 min at 525 ± 25 °C and cooled for 30–60 min in a desiccator, and then the weight was recorded. A total of 1 g of dried bamboo powder was weighed in crucible porcelain of known weight and put into a furnace at 525 ± 25 °C for 6 h. Afterward, it was removed from the furnace, cooled in a 30–60 min desiccator, and weighed.

#### 2.2.2. Degree of Crystallinity

The degree of crystallinity was estimated using X-ray diffraction (XRD) analysis diffraction intensity data. At 40 kV and 30 mA electrical current. An XRD (MaximaX-XRD 700, Shimadzu, Kyoto, Japan) was employed with a Cu K X-ray source (0.15406 nm). A small amount of sample (40–60 mesh particle size) was placed in a holder glass and evaluated at room temperature with a 2°/min scanning speed and a 10–40° scanning angle. As shown below, the degree of crystallinity was estimated using Equation (1) [27].

(1) $$Xc\;(\%)=\frac{Fc}{Fc+Fa}\times 100\%$$

where *Fc* denoted crystalline domains and *Fa* denoted amorphous domains.

With the assumption that the sharp and broad peaks related to crystalline and amorphous domains, the peaks (crystalline and amorphous) were fitted using the Lorentz function applied to the diffractograms.

#### 2.2.3. Near-Infrared Spectroscopy Acquisition

Powdered bamboo samples were divided into three parts i.e., bottom (B), middle (M), and top (T), and then they were analyzed using a Fourier transform near-infrared (NIR) spectrometer (Perkin Elmer Frontier, Waltham, MA, USA) to obtain chemical information based on NIR specific bands. Scanning for each part was conducted at a wavenumber of 10,000–4000 cm^−1^. The spectral resolution and scanning parameter was set to 16 cm^−1^ with 32 scans. The absorbance spectrum was recorded after the normalizing with the spectral as the background for the NIR machine [28]. After obtaining the original spectra, the second derivatives were made using a Savitzky–Golay method [29], smoothed at nine points, and in fifth polynomial order [30]. The script for second derivative spectra was written on Python programming 3.7 by the authors. The spectral region above 8000 cm^−1^ was excluded from this analysis due to the possibility of noises, and no significant information can be seen in this area [31]. The important chemical contents of belangke bamboos, such as lignin, extractives, cellulose, and hemicellulose, were analyzed based on the specific NIR band spectra by Schwanninger et al. [32].

#### 2.2.4. Physical and Mechanical Properties Tests of Bamboo

Test samples and procedures for physical (specific gravity and shrinkage) and mechanical (compression and shear strength) properties were made according to the ISO 22157-1:2019 standard [33].

- Specific gravity (SG)

Bamboo was cut into sizes of 2.5 cm × 2.5 cm and as thick as the wall thickness. Specific gravity of bamboo was calculated using Equation (2).

(2) SG=ODWV×1ρ

where *ODW* is oven-dry conditions (g), *V* is volume (cm^3^), and *ρ* is density of water at 4 °C (1000 kg/m^3^).

b. Shrinkage

**Figure 3:**
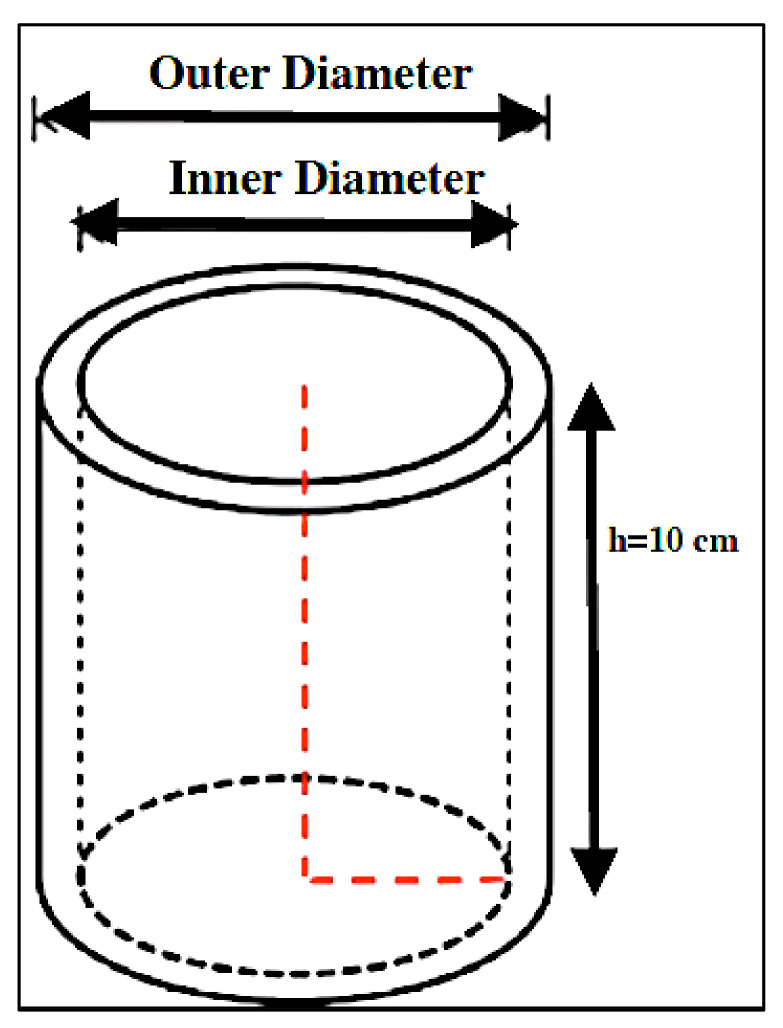
The sample used for the shrinkage test.

The test sample was made from a bamboo column with a height or length of 10 cm (Figure 3). In the shrinkage test, the parameters determined are outer diameter, inner diameter, and linear shrinkage. Samples were measured as initial conditions before drying (*I*). The sample was put into an oven with a temperature of 103 ± 2 °C and then measured again (*F*). The value of shrinkage was calculated according to the Equation (3):

(3) $$Shrinkage=\frac{I-F}{I}\times 100\%$$

where *I* is the initial reading and *F* is the final reading.

c. Compression strength test

**Figure 4:**
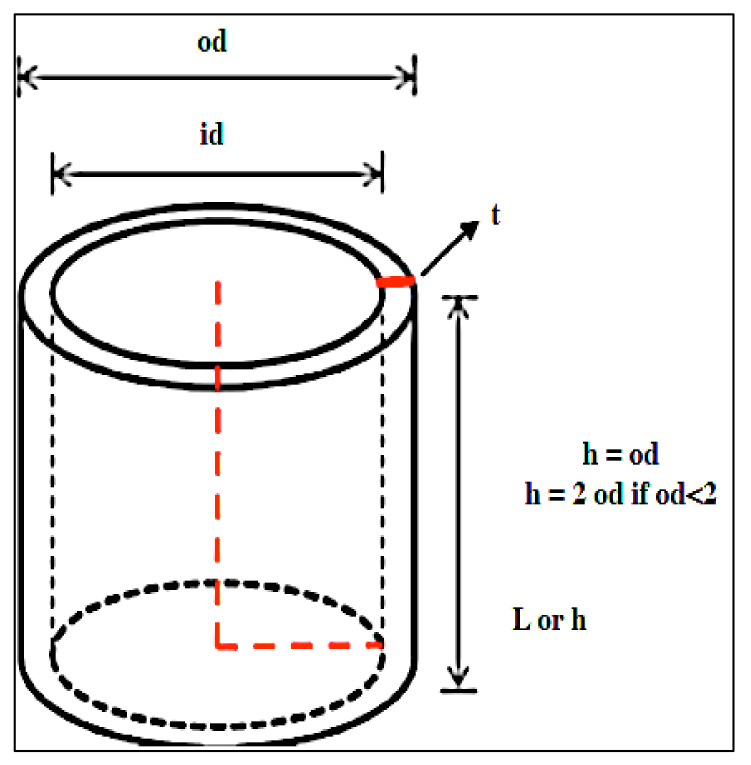
Sample used for the compression test.

The sample size of the compression test adjusts to the outer diameter of the bamboo. The height/length of the bamboo is the same as the outer diameter of the bamboo. For a bamboo diameter of less than 2 cm, the height of the bamboo sample is twice the outer diameter (Figure 4). The compressive strength was calculated using the Equation (4):

(4) $$\sigma_{ult}=\frac{F_{ult}}{A}$$

where *σ_ult_* is the ultimate compressive stress, in MPa, rounded off to the nearest 0.5 MPa; *F_ult_* is the maximum load at which the specimen fails, N; and *A* is the cross-sectional area, mm^2^.

d. Shear strength test

The sample size for the shear strength test adjusts to the outside diameter. Tests were performed parallel to the fibers. The height of the bamboo is equal to the outer diameter (Figure 4). The shear strength of bamboo can be calculated using Equation (5):

(5) $$\tau_{ult}=\frac{F_{ult}}{\sum \left(t\times L\right)}$$

where *τ_ult_* is the ultimate shear strength, MPa, rounded off to the nearest 0.1 MPa; *F_ult_* is the maximum load at which the specimen fails, N; and ∑(*t* × *L*) is the sum of the four products of *t* (wall thickness) and *L* (height).

e. Tension strength test

**Figure 5:**
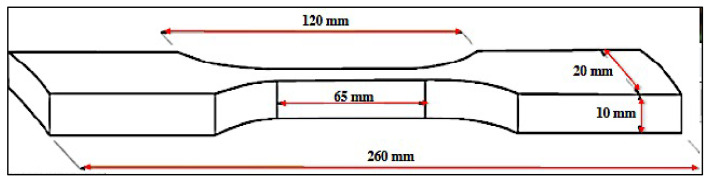
The sample used for the tension test.

The tensile strength test sample was prepared according to the ISO/TR 22157-2: 2004 standard [34]. The test sample is shown in Figure 5. The tensile strength was calculated using Equation (6):

(6) $$\sigma_{ult}=\frac{F_{ult}}{A}$$

where *σ_ult_* is the ultimate tension strength, MPa; *F_ult_* is the maximum load at which the specimen fails, N; and *A* is the volume of the sample (mm^3^) $A=\frac{\pi }{4}\times \left[D^{2}-{\left(D-2t\right)}^{2}\right]$.

#### 2.2.5. Particleboard

##### Material Preparation

Kemenyan wood (*Styrax sumatrana*) was converted into two types of particles: sawdust and wood shavings. Then, the belangke bamboo (*Gigantochloa pruriens*) was converted into a barkless strand. The particle sizes are presented in Table 1. The commercial adhesive used in this research was isocyanate type H3M with the specifications shown in Table 2. The isocyanate adhesive was produced by PT. Polychemie Asia Pacific (Jakarta, Indonesia).

The raw materials used, i.e., wood shavings, sawdust, and belangke bamboo, were placed in the oven until they reached a moisture content of 8%. In this work, three-layered particleboard (face, core, and back, at the ratio of 2:1:2) with dimensions of 250 mm × 250 mm, a thickness of 10 mm, and a target density of 750 kg/m^3^ were fabricated in the laboratory.

##### Particleboard Manufacturing

The dried particles were sprayed with 7% isocyanate adhesive. Meanwhile, for the surface layer (face and back), belangke bamboo strands were glued together using a brush in one of the surface areas. The bamboo strand as the back layer was arranged first into a mold measuring 250 mm × 250 mm, followed by the particles as the core layer. The next stage was laying the bamboo strand as a surface layer. The mattress was prepressed manually, then subjected to hot pressing in an automatically controlled laboratory hot press (custom by home industry in Bandung, Indonesia) at a temperature of 160 °C and pressure of 2.94 MPa for 5 min. The specifications of the laboratory hot press are presented in Table 3.

##### Physical and Mechanical Properties of Particleboard

**Figure 6:**
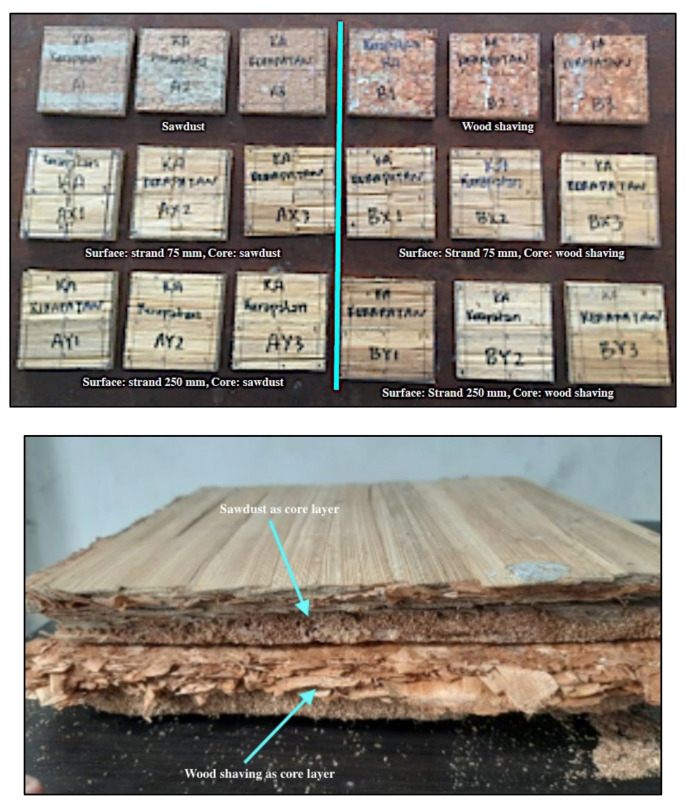
The appearance of coated and uncoated particleboard with belangke bamboo strands.

After hot pressing, the laboratory-fabricated particleboard panels (Figure 6) were conditioned for seven days in a conditioning room at a relative humidity of 65 ± 5% and 20 ± 2 °C prior to properties evaluation. The cutting of the test sample and evaluation of the boards’ physical and mechanical properties, i.e., density, moisture content, water absorption, thickness swelling, modulus of elasticity (MOE), modulus of rupture (MOR), and internal bond (IB), were performed according to the standard JIS A 5908 [35]. The sizes of the test samples used are presented in Table 4.

### 2.3. Data Analysis

The chemical components and physical–mechanical properties of bamboo were performed using a nonfactorial, completely randomized design. Chemical analysis was performed in duplicate, making a total of six-unit test samples. Meanwhile, for the physical and mechanical properties of bamboo, repeated testing of each test parameter was performed four times. The total test samples in this sub-research are twenty test sample units. The particleboard study used a factorial completely randomized design (CRD) model. The first factor is the type of particle (sawdust and shaving), and the second factor is the length of the bamboo strand as a coating material (75 mm and 250 mm). The number of replications for each treatment was three units. The total test samples in particleboard research are eighteen test sample units.

## 3. Results and Discussions

### 3.1. Chemical Components of Bamboo

#### 3.1.1. Structural Chemical Components of *Gigantochloa pruriens*

##### Acid Insoluble Lignin (AIL) and Acid Soluble Lignin (ASL)

**Figure 7:**
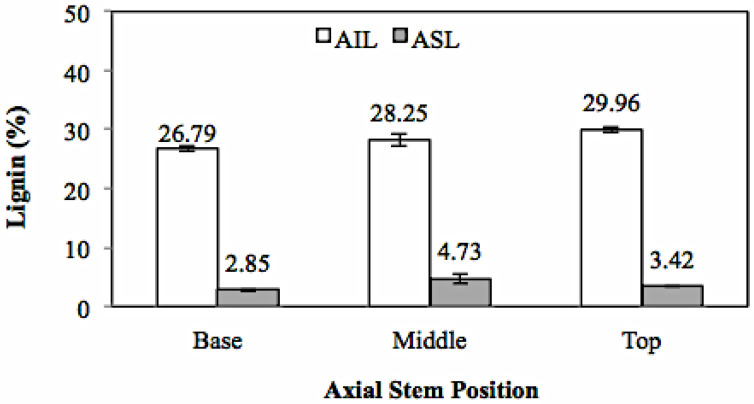
Lignin contents of *Gigantochloa pruriens*.

Lignin content is the total of AIL and ASL with the average value presented in Figure 7.

The AIL and ASL values ranged from 25.25–27.56% and 2.73–4.47%, respectively. The total lignin (AIL + ASL) content of *Gigantochloa pruriens* ranged from 27.97–30.79%, with the average value of the lignin content in all parts of the culm being 29.79%. Lignin in bamboo plants consists of p-coumaryl units with guaiacyl, syringyl, and p-hydroxyphenyl groups, which is similar to grasses or lignin in *Poaceae* plants [36].

The study related to the chemical components of bamboo performed by Li [37] reported that the lignin content of *Phyllostachys pubescens* ranged from 20–26%, while that of *Bambusa vulgaris* ranged from 11.6–20.3% [38]. Furthermore, Sharma et al. [39] reported that *P. pubescens* bamboo had a lignin content of around 22%. Loiwatu and Manuhuwa [40] reported that the lignin content of three types of bamboo, namely *Dendrocalamus asper*, *Schizostachyium brachycladum*, and *Schizostachyium lima*, was each 27, 37, 26, 18, and 26.05%. The lignin content of the species *Bambusa vulgaris* Schard var. vitata, *Gigantochloa nigrocillata*, *Gigantochloa apus*, *Gigantochloa verticillata*, and *Dendrocalamus asper*, obtained from Cibinong, West Java-Indonesia, varied between 30.01–36.88% [6].

Meanwhile, several researchers have reported the lignin content in other bamboo species from the *Gigantochloa* genus, including *Gigantochloa apus* with 22–25% [41] and *Gigantochloa brang*, *Gigantochloa levis*, *Gigantochloa Scortechinii*, and *Gigantochloa wrayi*—24%, 26%, 32%, and 26%, respectively [42]. In addition, Yusoff et al. [43] reported that the lignin content of *Gigantochloa albociliata* bamboo ranged from 22–24%. Based on the content of several *Gigantochloa* species, the average value of *Gigantochloa pruriens* lignin content is 32%, which is relatively higher compared to the other bamboo species. The high lignin content contributes to the high calorific value and structural rigidity, making it ideal for building components [5]. Statistical analysis showed that the axial position of the culm had no significant effect at the 95% confidence interval on the AIL (sig. 0.071) and ASL (sig. 0.054) values.

##### Holocellulose Content

**Figure 8:**
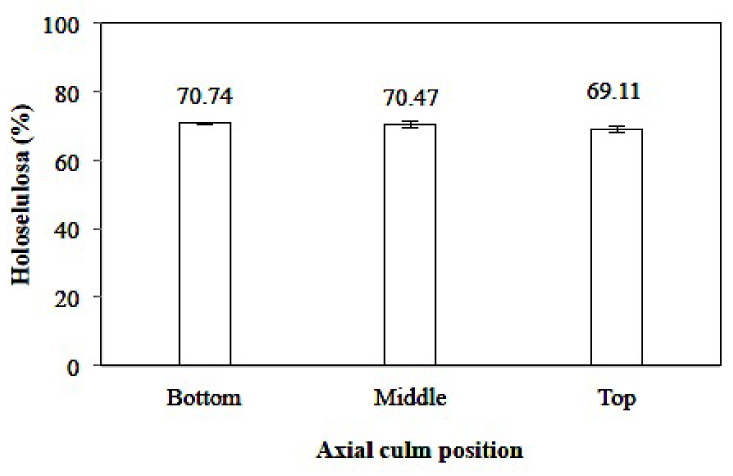
Holocellulose content of *Gigantochloa pruriens*.

The holocellulose content of *Gigantochloa pruriens* bamboo ranged from 63.56% to 66.66% (Figure 8), with the lowest and highest content being at the top and bottom of the culm, respectively. The difference in each part of the culm was also reported by Li [37], who determined that the culm’s position affects the amount of holocellulose content. *Gigantochloa pruriens* bamboo has holocellulose content equivalent to *Gigantochloa pseudoarundinacea* [44], which is slightly lower compared to *Dendrocalamus asper*, *Schizostachyium brachycladum*, *Schizostachyium lima* [40], and *Bambusa vulgaris* Var vulgaris [44].

Wahab et al. [42] obtained a holocellulose content value of 79%, 84%, 74%, and 84% for the bamboo species *Gigantochloa brang*, *Gigantochloa levis*, *Gigantochloa scortechinii,* and *Gigantochloa wrayi,* respectively. Yusoff et al. [43] reported that the holocellulose value of *Gigantochloa albociliata* ranged from 53–57%, higher than *Gigantochloa albociliata*. Statistical analysis showed that the axial position of the culm has no significantly different effect on the 95% confidence interval for holocellulose (sig. 0.239).

##### Alpha-Cellulose Content

**Figure 9:**
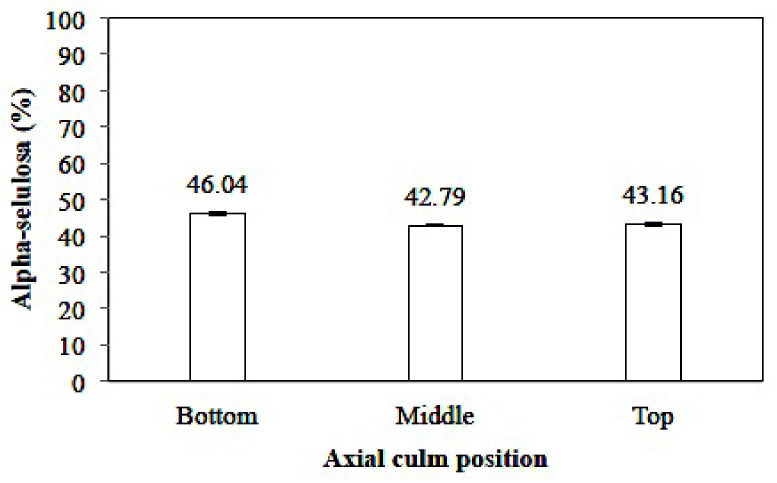
The alpha-cellulose contents of *Gigantochloa pruriens*.

A graphical representation of the alpha-cellulose content of *Gigantochloa pruriens* is given in Figure 9. The values ranged from 39.70–44.40%, with the lowest and highest holocellulose being in the top and at the bottom of the culm, respectively. The average value of alpha-cellulose in all parts of the culm was 41.48%, which falls within the normal range of alpha-cellulose (40–55%) as reported by Li [37]. This indicated that it was equivalent to *Schizostachyium brachycladum* but relatively lower compared to *Dendrocalamus asper* and *Schizostachyium lima*, as reported by Loiwatu and Manuhuwa [40]. However, it was still far below *Bambusa vulgaris*, as reported by Nahar and Hasan [45].

Li [37] stated that the range of cellulose content of bamboo was very suitable for its application as a raw material for the pulp and paper industry. Meanwhile, Nugroho et al. [46] reported that the parallel tensile strength of the bamboo fiber is quite highly correlated with the content of alpha-cellulose. Furthermore, it was stated that the mechanical properties and cellulose fraction in the cell wall play a greater role compared to the total polysaccharide content. Cellulose which has a linear polymer structure may affect the mechanical properties of wood, while more amorphous hemicellulose may affect the hygroscopic properties of bamboo.

Indriatie et al. [41] reported that *Gigantochloa apus* has an alpha-cellulose content of 69–72%, while Wahab et al. [42] reported that the α-cellulose content of *Gigantochloa* *brang*, *Gigantochloa* *levis*, *Gigantochloa* *scortechinii,* and *Gigantochloa* *wrayi* were 51, 33, 46, and 37%, respectively. Yusoff et al. [43] reported that the alpha-cellulose content of *Gigantochloa* *albociliata* ranged from 42–43%, which was relatively lower compared to *Gigantochloa* *apus, Gigantochloa brang*, and *Gigantochloa* *scortechinii*. The axial position of the culm had a significant effect on the 95% confidence interval for alpha-cellulose (sig. 0.005).

#### 3.1.2. Nonstructural Chemical Components of *Gigantochloa pruriens* Bamboo

##### Extractive Solubility in Ethanol Benzene (1:2)

**Figure 10:**
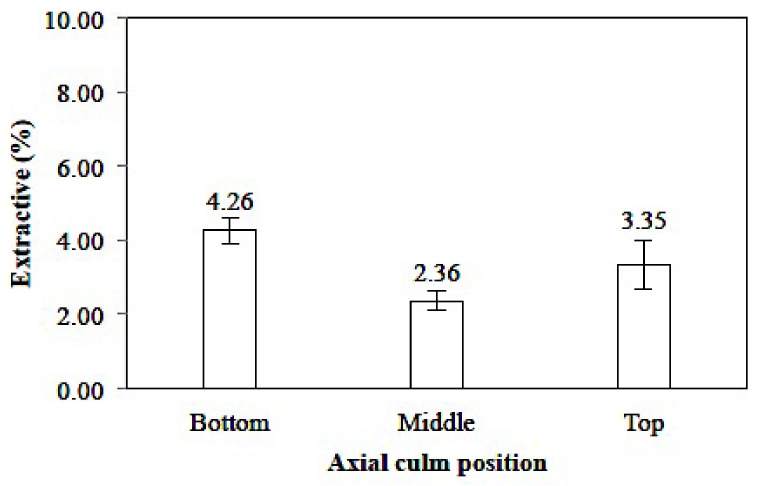
Extractive solubility in ethanol benzene (1:2) of *Gigantochloa pruriens*.

Extractives are nonpolymer organic materials soluble in nonpolar neutral organic solvents, such as alcohol-benzene, including hydrophobic oils, waxes, and resins [47]. The average value of the extractive solubility in benzene ethanol (1:2) of *Gigantochloa pruriens* bamboo is presented in Figure 10, ranging from 2.18–4.01%. The lowest and highest values were determined in the middle and at the bottom of the culm, respectively. This value is similar to that stated by Tolessa et al. [48], who state that the extractive content of *Oxytenanthera abyssinica* bamboo ranges from 3–5%.

The low extractive content is favorable for use in the pulp and paper industry [49]. The determined extractive content of *Gigantochloa pruriens* bamboo was relatively lower compared to *Gigantochloa* *apus* [41], *Gigantochloa brang*, *Gigantochloa levis*, *Gigantochloa scortechinii*, and *Gigantochloa wrayi* [42]. However, it was slightly higher compared to *Gigantochloa albociliata* bamboo [43].

The extractive content of *Gigantochloa pruriens* bamboo was different in each part of the culm. The extractive content of ethanol–benzene was less than 3–5%, contributing to several properties, such as color, odor, decay resistance, density, and flammability [48]. Extractive substances are a determining factor in the shrinkage properties of wood through physical and chemical mechanisms. These extractive substances play a vital role in wood stability as a bulking agent. Furthermore, they play a chemical role through the hydrophobic nature of the compounds contained in extractive substances. It is generally nontoxic, therefore enabling the preservation of all bamboo species before use because of their susceptibility to be attacked by powdery mildew, fungi, and termites compared to wood [40]. The higher alcohol–toluene extractive content in bamboo may be useful as an anti-rot agent and provides good strength due to its higher specific gravity. Generally, the higher solubility of tannins, gums, sugars, starches, and dyes indicates easier access and penetration of chemicals into cell walls [48]. Statistical analysis showed that the axial position of the culm has a significantly different effect on the 95% confidence interval on the solubility of the extractive in benzene–ethanol (sig. 0.055).

##### Ash Contents

**Figure 11:**
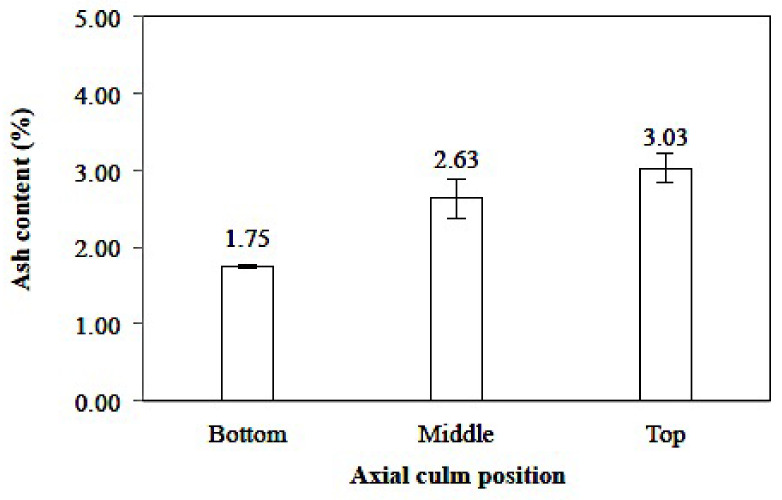
Ashes content based on *Gigantochloa pruriens*.

The average value of the ash content of *Gigantochloa pruriens* bamboo ranged from 1.36 to 2.57%, with an increase in ash content from the position of the bottom of the culm to the top, as seen in Figure 11. Ashes may be identified due to unburned compounds containing calcium, potassium, magnesium, manganese, and silicon.

The average value of ash content in all culms was 2%, while the ash content of *Gigantochloa brang*, *Gigantochloa levis*, *Gigantochloa scortechinii,* and *Gigantochloa wrayi* are 1.2; 1.3; 2.8; and 0.8% [42], respectively. Yusoff et al. [43] reported that the ash content of *Gigantochloa albociliata* ranged from 1.5–1.8%. Meanwhile, that of bangkele bamboo is equivalent to *Gigantochloa apus* (2.45%) and *Gigantochloa levis* (2.45%) but lower compared to that of *Gigantochloa atroviolacea* (3.29%) [50]. However, it is relatively higher than *Gigantochloa brang*, *Gigantochloa levis*, *Gigantochloa scortechinii,* and *Gigantochloa albociliata.* The axial position of the culm had a significantly different effect on the 95% confidence interval on the ash content of *Gigantochloa pruriens* bamboo (sig. 0.013).

### 3.2. Degree of Crystallinity

**Figure 12:**
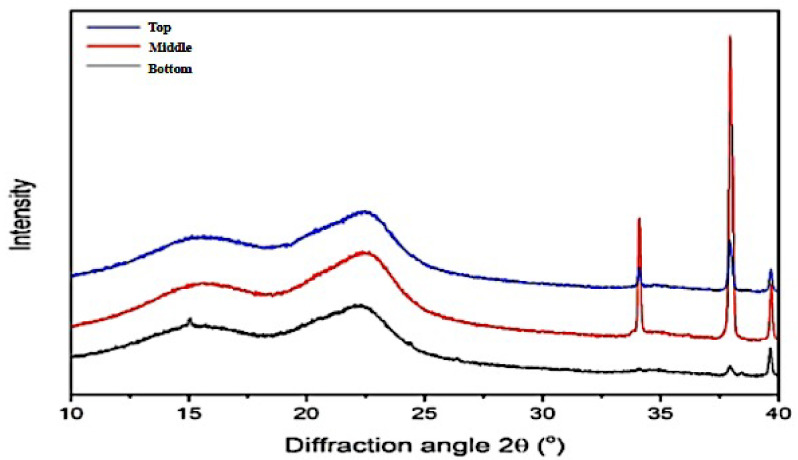
X-ray diffractograms of *Gigantochloa pruriens* bamboo based on axial position.

The crystallinity of woody plants is the crucial factor for responding to the properties of tree growth, anatomical structure, and chemical characteristics. Furthermore, it also has a great effect on Young’s modulus, dimensional stability, density, and wood hardness [51]. The degree of crystallinity of *Gigantochloa pruriens* bamboo is based on axial position and was calculated based on the relative amounts of crystalline and amorphous parts in the bamboo (Table 5). The X-ray diffractogram of bamboo in the top, middle, and bottom had a similar pattern as shown in Figure 12, with the highest peak in the middle position as the present Cellulose I structure with a monoclinic crystal structure. This structure is commonly found in plants, including bamboo. Although the alpha-cellulose content of bamboo in the middle position was the lowest, the degree of crystallinity in the middle position was the highest. It can be influenced by the difference in chemical composition in each axial position. There is no direct correlation between the cellulose content of hemicellulose and the lignin content of bamboo with the degree of crystallinity. A similar report was also found to have no significant variation in crystallinity in the longitudinal direction within each internode of bamboo. Further observation is needed to ensure the reason for the relationship between bamboo growth and lignification and chemical composition [52]. Lignin and hemicellulose can be included in the amorphous part (2θ of 15–22°), while the crystalline part is derived from cellulose with 2θ of around 22° [51,53,54]. Compared to a previous report on betung bamboo [55], which found that the degree of crystallinity was 30.43%, the degree of crystallinity In the bottom was higher than the value. This degree of crystallinity is lower than that of acacia and pine wood by as much as 44.10% and 35.73%, respectively [56]. The variation in the degree of crystallinity based on the axial position in *Gigantochloa pruriens* bamboo had a similar tendency to the variation in chemical composition in the axial position of raru wood [57]. In addition to affecting the chemical properties, the degree of crystallinity also affects the mechanical properties in this study. The high degree of crystallinity in the middle of the bamboo belangke culm is shown to cause the tensile strength value to be higher than the other culm positions. Barrios et al. [58] and Takeuchi et al. [59] state that the degree of crystallinity positively correlates with dimensional stability and tensile strength. Furthermore, Li et al. [60] also reported that a high degree of crystallinity in the outer wood of *Pinus radiata* caused an increase in the value of MOR, MOE, and compression parallel to the fiber.

### 3.3. Near-Infrared Analysis

**Figure 13:**
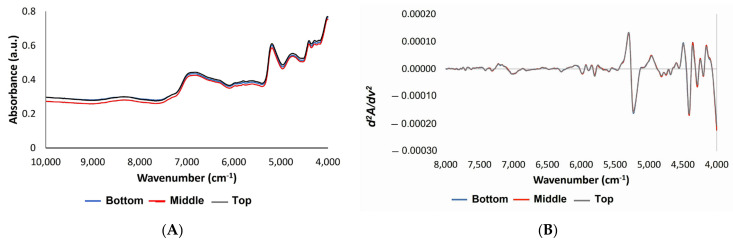
The original spectra of NIR at the wavenumber 10,000–4000 cm^−1^ (**A**); second derivative spectra at the wavenumber 8000–4000 cm^−1^ (**B**).

A graphical representation of the original (A) and second derivative spectra (B) of belangke bamboo in the axial direction is given in Figure 13. At first glance, the original spectra of belangke bamboo in the axial direction (Figure 13A) were almost similar, thus the important bands associated with major chemical components of bamboo were not well resolved. These spectra have only visualized the discrepancies in baseline level [61], where the top part of the bamboo was slightly higher than other regions. Via et al. [62] reported that density was one of the factors that affected the absorbance of NIR spectra. The higher density corresponded to a higher absorbance level. Typically, the bamboo’s top is higher than its other portions [56].

On the contrary, second derivative spectra (Figure 13B) visualized more different bands than the original ones [63]. In addition, these spectra revealed some important bands of the chemical components [28], thus analyzing the chemical contents, such as lignin, cellulose, holocellulose, and extractives, which were based on these spectra. The second derivative spectra were divided into four regions based on the characteristics of the chemical functional groups: the first region 10,000–7300 cm^−1^ were second or third overtones, but there was less information on wood components; the second region 7300–6050 cm^−1^ were OH overtone vibrations; the third range 6050–5500 cm^−1^ were CH vibrations and the vibrations from aromatic structure; and the fourth region 5500–4000 cm^−1^ were several combinations of vibrations and coupling vibrations [28,32]. Although the NIR second derivative spectra of belangke bamboo were almost identical, some peak regions had different characteristics in the axial direction of the stem.

**Figure 14:**
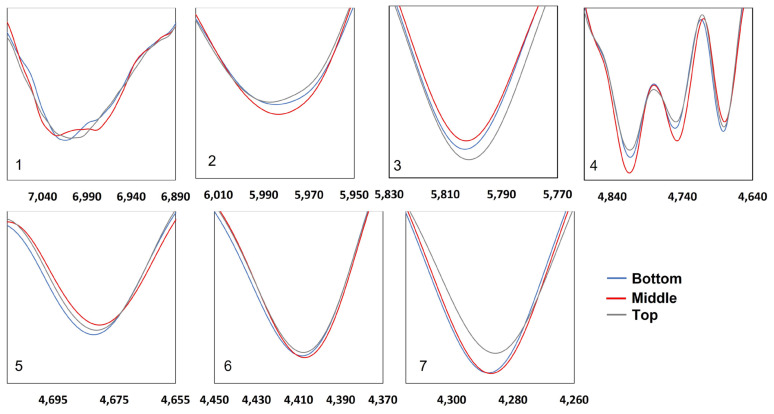
Enlarged bands at specific regions of second derivative spectra: (**1**) bands 7000 cm^−1^ assigned to an amorphous region of cellulose; (**2**) bands 5980 cm^−1^ assigned to aromatic skeletal due to lignin; (**3**) bands 5800 cm^−1^ assigned to furanose or pyranose due to hemicellulose; (**4**) bands 4890–4,620 cm^−1^ assigned to cellulose region; (**5**) bands 4686 cm^−1^ assigned to lignin or extractives; (**6**) bands 4404 cm^−1^ assigned cellulose and hemicellulose; and (**7**) bands 4283 cm^−1^ assigned to cellulose, hemicellulose, and xylan.

Therefore, this study analyzed the peak characteristics of the NIR spectra of belangke bamboo, which are assigned to the main chemical components, such as cellulose, holocellulose, lignin, and extractives, through enlarging the band regions on the second derivative spectra (Figure 14). These bands were mainly located in the third range for lignin and the fourth range for cellulose. According to the NIR second derivative spectra, the band assigned to holocellulose, such as 4404 cm^−1^ (cellulose and hemicellulose), was identical. This result corresponded to the wet chemical analysis for holocellulose in terms that the values were not significantly different based on the axial direction. Markedly, bands 4283 cm^−1^ assigned to cellulose, hemicellulose, and xylan seem slightly different with the bottom part having the lowest peak. In the band at 4808 cm^−1^ at the semi-crystalline and crystalline regions as well as 4739 cm^−1^ (cellulose) at the cellulose group region, the middle part had the lowest value. It was assumed that this region had a higher degree of crystallinity, as demonstrated by the positive correlation with the results of the XRD analysis. The extractive content with the bands at 4686 cm^−1^ showed that the bottom part was supposed to have high extractive content while the band at 5980 cm^−1^ showed that the middle part tended to have more lignin content than another part. However, the wet chemical analysis revealed that there were nonsignificant values of lignin content at the different high levels of the bamboo stem.

### 3.4. Physical and Mechanical Properties of Bamboo

#### 3.4.1. Physical Properties of Bamboo

A summary of the physical properties of belangke bamboo is presented in Table 6.

##### Specific Gravity (SG)

The SG value of belangke bamboo ranged from 0.58 to 0.60. The bottom had a higher SG value than the middle, which was confirmed by its chemical components where this bamboo had a higher alpha-cellulose content value at the bottom. High SG indicates a higher proportion of cell walls and cellulose content, which will affect the mechanical properties, especially the MOR value, as stated by Baharoǧlu et al. [64]. The SG value of belangke bamboo was equivalent to that of apus bamboo (*Gigantochloa apus*), as reported by Abdullah et al. [65]. Analysis of variance on the SG value showed that the axial position of the bamboo culm was not significantly different at the 95% confidence level (sig. 0.696).

##### Shrinkage

The inner diameter, outer diameter, and linear shrinkage ranged from 0.94–3.67%, 1.29–3.17%, and 0.13–0.21%, respectively. The shrinkage of the outer diameter was greater than the inner diameter due to the denser vascular bundle diameter on the outside of the bamboo [14]. The effect of density is evident in this shrinkage parameter. The greater the density, the greater the shrinkage value of the bamboo. Furthermore, the linear shrinkage tends to increase from the bottom to the top of the bamboo stem. Darwis et al. [14] reported that the percentage of belangke bamboo vascular bundles at the bottom of the culm was lower than at the middle and top. The high proportion of vascular bundles impacts the amount of bamboo shrinkage. Analysis of variance on the inner and outer shrinkage values showed that the axial position of the bamboo culm was not significantly different at the 95% confidence level. At the same time, linear shrinkage was not significantly different.

#### 3.4.2. Mechanical Properties of Bamboo

A summary of the results obtained for the mechanical properties of belangke bamboo is presented in Table 7.

##### Compression Strength

The compressive strength values ranged from 38.96–45.44 MPa. The highest value was determined at the bottom of the bamboo culm, and the lowest value was obtained at the top. The compressive strength value of belangke bamboo decreased from the bottom to the top. Darwis et al. [14] reported that the number of vascular bundles in belangke bamboo at the position of the bottom of the stem was the lowest. Chowdhury et al. [66] stated that the fiber’s compression value was negatively correlated with fiber diameter and vessel cells but positively correlated with vessel wall thickness. Analysis of variance on compression strength value showed that the axial position of the bamboo culm was not significantly different at the 95% confidence level (sig. 0.396).

##### Tensile Strength

The tensile strength values ranged from 95.88 to 278.74 MPa, the highest value was in the middle of the bamboo culm, and the lowest was at the top. According to Epsiloy [67], fiber length affects the mechanical properties of bamboo. Darwis et al. [14] reported that the middle part of the belangke bamboo culm has longer fibers than the bottom and ends of the bamboo, so it has the highest tensile strength value. In addition to differences in anatomical structure, the chemical components that comprise bamboo can also cause these differences. Lignin and alpha-cellulose significantly affect the tensile strength of bamboo. Lignin contributes to the stiffness properties, while cellulose has a linear polymer structure with a high crystalline fraction. The results showed that the center of the bamboo culm had the highest crystallinity value. Analysis of variance on the tensile strength value showed that the axial position of the bamboo culm was significantly different at the 95% confidence level (sig. 0.000).

##### Shear Strength

The value of shear strength ranged from 7.39 to 7.79 MPa; the highest value for shear strength was at the top of the bamboo culm, and the lowest was at the bottom. It was attributed to the presence of belangke bamboo parenchyma. The proportion of vascular bundles and parenchyma as the matrix affects the shear strength of bamboo. When the number of proportions of the matrix gets bigger, the tendency to decrease the shear strength gets bigger. Darwis et al. [14] reported that the bottom of the belangke bamboo culm has the largest parenchyma. The cause of the low shear strength at the bottom is also due to the low lignin content. Longui et al. [68] stated that lignin is proven to be able to increase the stiffness properties of the secondary walls and cohesion between wood tissues and has a strong effect on the mechanical properties of wood, especially in the transverse direction. Analysis of variance on the shear strength value showed that the axial position of the bamboo culm was not significantly different at the 95% confidence level (sig. 0.874).

### 3.5. Physical and Mechanical Properties of Particleboard

A summary of the physical and mechanical properties of the laboratory-fabricated three-layered particleboards, manufactured with belangke bamboo as a reinforcing material, is given in Table 8.

#### 3.5.1. Density

As seen from the results in Table 8, the average values of particleboard density ranged from 620–690 kg/m^3^. The lowest value was found on the B2 type board, while the highest value was obtained for the A0 type board. The density of the laboratory-produced boards was below the targeted value of 750 kg/m^3^. This might be attributed to: (1) the loss in particles during the mat-forming and compression processes following the statement of Bufalino et al. [69] that the low value of particleboard density is due to the presence of some particles that are wasted during the manufacturing process; (2) spring back, in which the average spring back value in this study was almost 30%, meaning that the board after conditioning experienced a thickness swelling of nearly 1/3 of the target thickness of 1 cm. Several other factors that affect the density value of particleboard include the density of wood, compression pressure, and the amount of adhesive used [70]. Bowyer et al. [71] stated that the density value is highly dependent on the wood density and compression pressure.

Analysis of variance on the density value showed that the interactions between particle types and strand length are not significantly different at the 95% confidence level (sig. 0.070). Overall, the density value of this particleboard has met the JIS A 5908-2003 standard, which requires the board density value to be between 400–900 kg/m^3^.

#### 3.5.2. Moisture Content (MC)

According to Table 7, the moisture content values of the particleboard ranged from 6.56–7.96%. The lowest value was found for the B2 type board, while the highest value was determined for the A0 type board. Particleboard with sawdust core layer had a higher moisture content than the wood shavings core layer. Farrokhpayam et al. [72] reported that the fine particle size would absorb water vapor more significantly than the coarse particle size. Using bamboo strand as a coating on the board’s surface tends to reduce the board’s MC. Analysis of variance on the MC value showed that the interactions between particle types and strand length are not significantly different at the 95% confidence level (sig. 0.107). Overall, the MC of this particleboard met the JIS A 5908-2003 standard requirements, i.e., MC values between 5–13%.

#### 3.5.3. Water Absorption (WA)

The WA value of the board ranges from 21.72–68.41%. The lowest value was found for the B0 type board, while the highest one was determined for the A2 type board. A similar trend with the MC parameter also occurs in the WA parameter. A board with a smaller particle size results in a relatively higher WA value than a board with larger particles. Sawdust has a greater high-surface area per unit weight compared to that of wood shaving; therefore, it tends to absorb more water [19]. In this study, using bamboo strands as a surface layer resulted in increased WA value compared to the uncoated boards—density is a factor that affects the board’s WA value. A higher board density causes a lower board WA value. Bufalino et al. [69] stated that the density value and adhesive content affect water absorption. Meanwhile, Vital et al. [73] noted that the board density value is the opposite of the WA value. Analysis of variance on the WA value showed that the interactions between particle types and strand length are significantly different at the 95% confidence level (sig. 0.000).

#### 3.5.4. Thickness Swelling (TS)

Thickness swelling values ranged from 4.38–9.57%. The lowest value was determined for the A0 type board, while the highest value was obtained for the B2 type board. Using bamboo strands as a surface layer increases the board’s TS value. It is related to the density of the panels produced in this study. Strand-coated boards on the surface tend to have lower density values. The board with belangke bamboo strands on the surface exhibited a spring back of 60% higher than the control. Iswanto et al. [74] reported the effect of relatively high spring back as an indicator of the cause of the high TS value of the sorghum stem particleboard coated with rope bamboo strand (*Gigantochloa apus*). The high spring back of the board is thought to be due to the weak bond between sawdust or wood shavings and bamboo. The inhomogeneity of these two types of particles is a weak point, resulting in the board’s high spring back value being coated with bamboo strands on the surface.

Overall, boards with a core layer of sawdust had a lower TS value than wood shavings. Farrokhpayam et al. [72] reported that the finer particle size resulted in a lower TS value of the board than the coarser or larger particle size. Analysis of variance on the TS showed that the interaction between particle types and strand length are not significantly different at a 95% confidence interval for the resulting particleboard thickness swelling value (sig. 0.531). Markedly, the particleboard panels fabricated in this work met the JIS A 5908-2003 standard requirements of a maximum thickness swelling of 12%.

#### 3.5.5. Modulus of Elasticity (MOE) and Modulus of Rupture (MOR)

**Figure 15:**
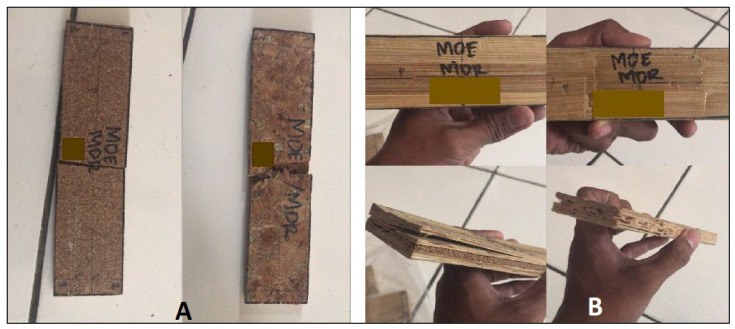
The appearance of damage in samples in MOE and MOR testing: nonstrand-coated board (**A**), and strand-coated board (**B**).

The average MOE value of the laboratory-fabricated particleboards ranged from 0.32–13.63 GPa, and the MOR value ranged from 6.39–48.37 MPa. The lowest MOE value of 0.3 GPa was determined for the A0 board, while the highest value of 13.6 GPa was obtained for the B1 board. Regarding the MOR, the lowest value of 6.4 MPa was determined for the A0 particleboard, and the highest value of 48.4 MPa was obtained for the B2 board. The appearance of damage in the test specimen in bending tests is presented in Figure 15.

**Figure 16:**
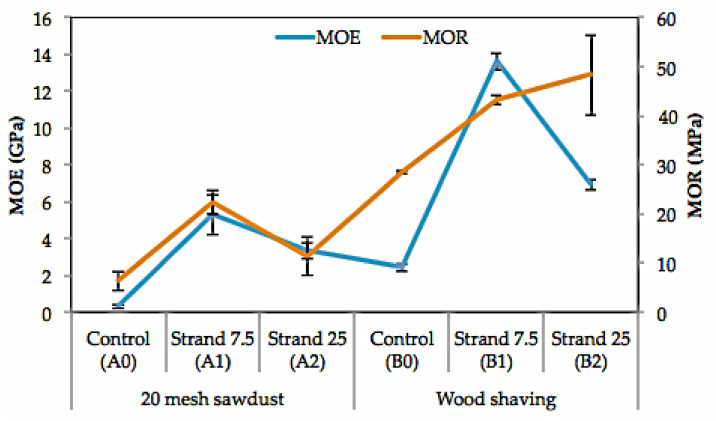
Modulus of rupture (MOR) and modulus of elasticity (MOE) of particleboard fabricated in this work.

Overall, using belangke bamboo strands as a surface layer resulted in higher MOE and MOR values compared to uncoated boards. The use of belangke bamboo as a coating can increase the MOE and MOR values by 16 and 3 times, respectively (Figure 16). This is due to the good density and flexural strength of bamboo. Rofii et al. [75] stated that high-density materials in the surface layer obtained better curvature than materials with low specific gravity. Iswanto et al. [9] reported that using the belangke bamboo strand as a surface layer on particleboard resulted in higher MOE and MOR values than Tali (*Gigantochloa apus*) bamboo strand because the specific gravity of belangke bamboo was higher than that of *Gigantochloa apus*. According to Iswanto et al. [74], boards coated with bamboo strands have higher flexural strength than boards coated with thin plywood. Using bamboo as a coating material on the surface layer has a positive effect on increasing mechanical properties, especially flexural strength and fracture strength, as reported by Iswanto et al. [9,74].

Overall, the use of larger particles, in this case, shavings, showed higher MOE and MOR values when compared to powders [76,77,78,79]. This condition is in line with the research conducted by Farrokhpayam et al. [72], who reported that fine-sized particles have lower MOE and MOR values than medium- and coarse-sized particles. Wood shavings have a high slenderness ratio value when compared to powder. Badejo [80] and Rokiah et al. [81] stated that strands or particles that are longer and thinner would produce higher MOR values compared to short and thick sizes.

Analysis of variance on the MOE and MOR values showed that the interaction between particle type and strand length are significantly different at a 95% confidence level (sig. 0.000). Overall, the MOE and MOR values of the particleboard met the JIS A 5908-2003 standard requirements of a minimum of 2.05 GPa and 7.85 MPa, respectively.

#### 3.5.6. Internal Bond (IB)

The IB values of the boards ranged from 0.15–0.39 MPa. The lowest value was found for the B1 type board, while the highest value was obtained for the A0 type board. The laboratory boards with surfaces coated with bamboo strands exhibited lower IB values. This was attributed to the weak particle bonds between the surface layer and the core layer due to the use of two different types of particles.

Overall, the core layer’s finer particle size (powder) resulted in higher IB values than the shavings particles. Cosereanu et al. [82] stated that fine particles with a flat surface and diffuse appearance could achieve better bond contact so that the structure obtained was more compact and homogeneous. Meanwhile, coarse particles with localized concave geometry produce adhesive agglomeration and reduce compactness, thereby reducing internal bonding strength.

The density of the boards also affects the IB values. In this study, the core layer in the form of sawdust resulted in a higher board density than the type of shavings particles. Wong et al. [83] stated a linear relationship between the board density and IB value. Warmbier et al. [84] reported that IB and screw holding strength increased with increasing board density values.

Analysis of variance on the IB value showed that the interaction between particle type and strand length are significantly different at a 95% confidence level (sig. 0.027). Overall, the resulting IB value met the JIS A 5908-2003 standard, which requires a minimum value of 0.15 MPa.

## 4. Conclusions

From this study, it was observed that the lignin content of *Gigantochloa pruriens* bamboo includes AIL and ASL, with the former having the lowest and highest values at the bottom and top of the culm, respectively (25.25–27.56%). Meanwhile, ASL had the lowest and highest values at the bottom and middle of the culm (2.73–4.47%), respectively, with the lowest holocellulose content being at the top of the culm and the highest at the bottom (63.56–66.66%). Furthermore, it has the lowest alpha-cellulose in the top and the highest at the bottom (39.70–44.40%). The lowest extractive solubility in ethanol–benzene (1:2) was at the middle culm position and the highest at the bottom (2.18–4.01%). Meanwhile, the lowest ash content was determined in the bottom of the culm and the highest at the top, respectively (1.36–2.57%). The crystallinity degree of *Gigantochloa pruriens* bamboo in the axial position varied between 29.78–37.95%. In this work, the degree of crystallinity also influences mechanical and chemical properties of bamboo. 

The physical properties of bamboo, including density, inner and outer diameter shrinkage, and linear shrinkage, were 0.59, 2.18%, 2.26%, and 0.18%, respectively. Meanwhile, bamboo’s mechanical properties, including compressive strength, shear strength, and tensile strength, were 42.19 MPa, 7.63 MPa, and 163.8 MPa, respectively. Based on its density, belangke bamboo was assigned to strength class III.

The study demonstrated the potential of using belangke bamboo strands as a surface coating material in particleboard manufacturing. Despite having higher WA and TS values compared to the uncoated counterparts, particleboard panels reinforced with belangke bamboo strands demonstrated satisfactory mechanical strength. Markedly, the addition of belangke bamboo strands as a reinforcing material in particleboards significantly improved the mechanical properties of the boards, resulting in increased MOE and MOR values of particleboards by 16 and 3 times, respectively.

## Figures and Tables

**Table 1:** The particle size of *Styrax sumatrana* wood and *Gigantochloa pruriens*.

Particle Type	Length (cm) *	Width (cm) *	Thickness (cm) *	Mesh	Slenderness Ratio
Sawdust	-	-	-	20	-
Shaving	35.26 (11.44)	18.66 (5.76)	0.14 (1.08)	N/A	252.02 (35.09)
Strand 7.5 cm	7.50 (0.03)	2.51 (0.03)	0.13 (0.02)	N/A	62.52 (8.25)
Strand 25 cm	25.34 (0.19)	2.79 (0.40)	0.09 (0.25)	N/A	297.69 (91.73)

Remark: (*) the number of measurement samples is 100 units. Values in parentheses represent the standard deviation.

**Table 2:** Properties of isocyanate adhesive used in this work.

Properties	Information
Solids content	98%
Viscosity	150–250 cps/23 °C
pH	6.5–8.5
Pot life mixture	Within 60 min
Spread volume	200~250 g/m^2^
Assembly time	Within 10 min
Cold press for a low-density wood	0.69–0.98 MPa
Cold press for a high-density wood	0.98–1.47 MPa
Press time Cold Press	30–45 min depends on wood species, size of lamella, temperature, and spread volume

Source: PT. Polychemie Asia Pasific (Jakarta, Indonesia).

**Table 3:** Specification of the hot press used.

No	Specification	
1	Height	160 cm
2	Length	90 cm
3	Width	50 cm
4	Pressure plate area	35 × 35 cm^2^
5	Maximum hydraulic pressure	20.59 MPa
6	Hydraulic lifting	100 ton
7	Maximum heating power	2 × 3000 watt
8	Maximum temperature	250 °C

**Table 4:** Particleboard test sample sizes.

Parameter	Size
Density	10 cm (length) × 10 cm (width)
Moisture content (MC)	10 cm (length) × 10 cm (width)
Water absorption (WA)	5 cm (length) × 5 cm (width)
Thickness swelling (TS)	5 cm (length) × 5 cm (width)
Modulus of elasticity (MOE)	20 cm (length) × 5 cm (width)
Modulus of rupture (MOR)	20 cm (length) × 5 cm (width)
Internal bond (IB)	5 cm (length) × 5 cm (width)

**Table 5:** Degree of crystallinity of *Gigantochloa pruriens* bamboo based on the axial position.

Sample	*Fc* (kcps.deg)	*Fa* (kcps.deg)	*Xc*%
Bottom	22.8276	46.5733	32.89
Middle	41.598	68.0017	37.95
Top	26.567	62.6533	29.78

**Table 6:** Physical properties of *Gigantochloa pruriens*.

Physical Properties	Bottom	Middle	Top
Specific gravity	0.60 (0.08)	0.58 (0.03)	0.60 (0.03)
Outer Diameter Shrinkage (%)	2.33 (0.45)	1.29 (0.45)	2.33 (0.10)
Inner Diameter Shrinkage (%)	1.94 (0.62)	0.94 (0.62)	3.67 (0.14)
Linear Shrinkage (%)	0.13 (0.07)	0.2 (0.04)	0.21 (0.09)

Values in parentheses are standard deviation.

**Table 7:** Mechanical properties of *Gigantochloa pruriens*.

Mechanical Properties	Bottom	Middle	Top
Compression Strength (MPa)	45.44 (0.66)	42.15 (6.63)	38.96 (8.84)
Tensile Strength (MPa)	116.77 (20.90)	278.74 (15.20)	95.88 (5.46)
Shear Strength (MPa)	7.39 (0.61)	7.69 (1.59)	7.79 (0.94)

Values in parentheses are standard deviation.

**Table 8:** Physical and mechanical properties value of particleboard produced in this work. Particleboard made with 100% wood sawdust were denoted as A0 while those reinforced with 75 mm and 250 mm bamboo strands were denoted as A1 and A2, respectively. Meanwhile, particleboard made with 100% wood shavings were denoted as B0 while those reinforced with 75 mm and 250 mm bamboo strand were denoted as B1 and B2.

Parameter	20 Mesh Sawdust			Wood Shaving		
	Control (A0)	Strand 7.5 (A1)	Strand 25 (A2)	Control (B0)	Strand 7.5 (B1)	Strand 25 (B2)
Density (kg/m^3^)	690 (30)	640 (30)	680 (40)	670 (10)	670 (30)	620 (10)
MC (%)	7.96 (0.25)	7.10 (0.18)	6.89 (0.37)	7.10 (0.46)	6.66 (0.17)	6.56 (0.30)
WA (%)	47.70 (5.99)	45.94 (8.83)	68.41 (6.73)	21.72 (5.39)	31.46 (6.72)	46.12 (3.60)
TS (%)	4.38 (0.77)	6.50 (1.56)	7.37 (1.38)	5.29 (0.86)	7.01 (1.05)	9.57 (2.42)
MOE (GPa)	0.32 (0.12)	5.28 (1.08)	3.34 (0.45)	2.41 (0.19)	13.63 (0.47)	6.91 (0.27)
MOR (MPa)	6.39 (1.99)	22.38 (2.40)	11.38 (3.88)	28.52 (0.38)	43.17 (0.89)	48.37 (8.19)
IB (MPa)	0.39 (0.09)	0.19 (0.04)	0.29 (0.08)	0.29 (0.04)	0.15 (0.04)	0.18 (0.01)

Values in parentheses are standard deviation.

## Data Availability

The data presented in this study are available on request from the corresponding author.
